# The Growth Suppressing Effects of Girinimbine on Hepg2 Involve Induction of Apoptosis and Cell Cycle Arrest

**DOI:** 10.3390/molecules16087155

**Published:** 2011-08-23

**Authors:** Suvitha Syam, Ahmad Bustamam Abdul, Mohd. Aspollah Sukari, Syam Mohan, Siddig Ibrahim Abdelwahab, Tang Sook Wah

**Affiliations:** 1 UPM-Makna Cancer Research Laboratory, Institute of Bioscience, Universiti Putra Malaysia, 43400 Serdang, Selangor, Malaysia; 2 Department of Chemistry, Faculty of Science, Universiti Putra Malaysia, 43400 Serdang, Selangor, Malaysia; 3 Centre for Natural Products and Drug Discovery (CENAR), Department of Pharmacology, Faculty of Medicine, University of Malaya, 50603 Kuala Lumpur, Malaysia; 4 Department of Pharmacy, Faculty of Medicine, University of Malaya, 50603 Kuala Lumpur, Malaysia

**Keywords:** anticancer, apoptosis, carbazole alkaloid, cell cycle arrest, girinimbine, pyranocarbazole

## Abstract

*Murraya koenigii* is an edible herb widely used in folk medicine. Here we report that girinimbine, a carbazole alkaloid isolated from this plant, inhibited the growth and induced apoptosis in human hepatocellular carcinoma, HepG2 cells. The MTT and LDH assay results showed that girinimbine decreased cell viability and increased cytotoxicity in a dose-and time-dependent manner selectively. Girinimbine-treated HepG2 cells showed typical morphological features of apoptosis, as observed from normal inverted microscopy and Hoechst 33342 assay. Furthermore, girinimbine treatment resulted in DNA fragmentation and elevated levels of caspase-3 in HepG2 cells. Girinimbine treatment also displayed a time-dependent accumulation of the Sub-G_0_/G_1_ peak (hypodiploid) and caused G_0_/G_1_-phase arrest. Together, these results demonstrated for the first time that girinimbine could effectively induce programmed cell death in HepG2 cells and suggests the importance of conducting further investigations in preclinical human hepatocellular carcinoma models, especially on *in vivo* efficacy, to promote girinimbine for use as an anticancer agent against hepatocellular carcinoma.

## 1. Introduction

Cancer represents one of the most deadly diseases in the World. Among the various types of cancer, hepatocellular carcinoma (HCC) is a common malignancy with high metastasis rates [1]. There is evidence suggesting that the incidence of HCC is rising all around the World [2,3]. The complexity of the disease advocates the need for the involvement of hepatologists, pathologists, radiologists, surgeons and oncologists in patient care [4]. The chances of recurrence of the disease are more than 70%, even after surgical resection [5]. Further, there is minimal survival rate with the systemic chemotherapeutic agents, which also have toxic effects [6]. Consequently there is an important need for new natural anticancer compounds in chemotherapeutics. 

**Figure 1:**
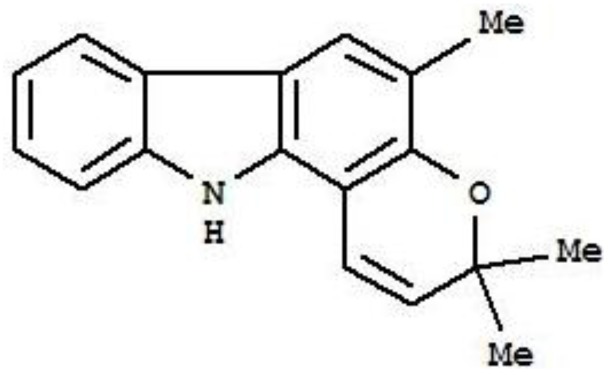
Structure of girinimbine (3,3,5-trimethyl-11H-pyrano[3,2-a]carbazole).

The plants of the genus *Murraya*, *Glycosmis* and *Clausena* from the Rutaceae family contain carbazole alkaloids [7]. These alkaloids possess various therapeutic capacities, including anti-tumor activity [8]. There are studies proving the anticancer potential of carbazole alkaloids against various cancer cell lines [8,9] and some of the carbazole alkaloids have entered clinical trials [9]. Girinimbine (3,3,5-trimethyl-11H-pyrano[3,2-a]carbazole, Figure 1) is a carbazole alkaloid, specifically a pyranocarbazole which was first isolated from the stem bark of *Murraya koenigii* [10]. Besides *M. koenigii*, it is also present in *Clausena dunniana* [11] and *Clausena heptaphylla* [12] from the Rutaceae family. The plant *M. koenigii* is readily available in most parts of Asia including India, Malaysia, China, and Sri Lanka and is used as a folk medicine [13]. The pyranocarbazole girinimbine has already drawn attention due to its wide range of pharmacological effects like anti-trichonomal [14] antiplatelet activity [15], antibacterial activity [16] and antitumor activity [17]. As an anticancer agent, girinimbine has proved to inhibit cell proliferation, produce cytotoxicity, and induce apoptosis [17,18]. Hence, the aim of this study was to evaluate the hypothesis that girinimbine may be a potential chemotherapeutic agent against HCC.

## 2. Results

### 2.1. Girinimbine Inhibits the Proliferation of HepG2 Cells

Since girinimbine has already been reported to have antiproliferative properties [17,18], we subjected HepG2 cells to girinimbine exposure and then evaluated the antiproliferative effects. To study the concentration- and time-dependent actions, we performed the treatment with six concentrations of girinimbine ranging between 1–400 µM for three different time periods; 24, 48 and 72 h each.

**Figure 2:**
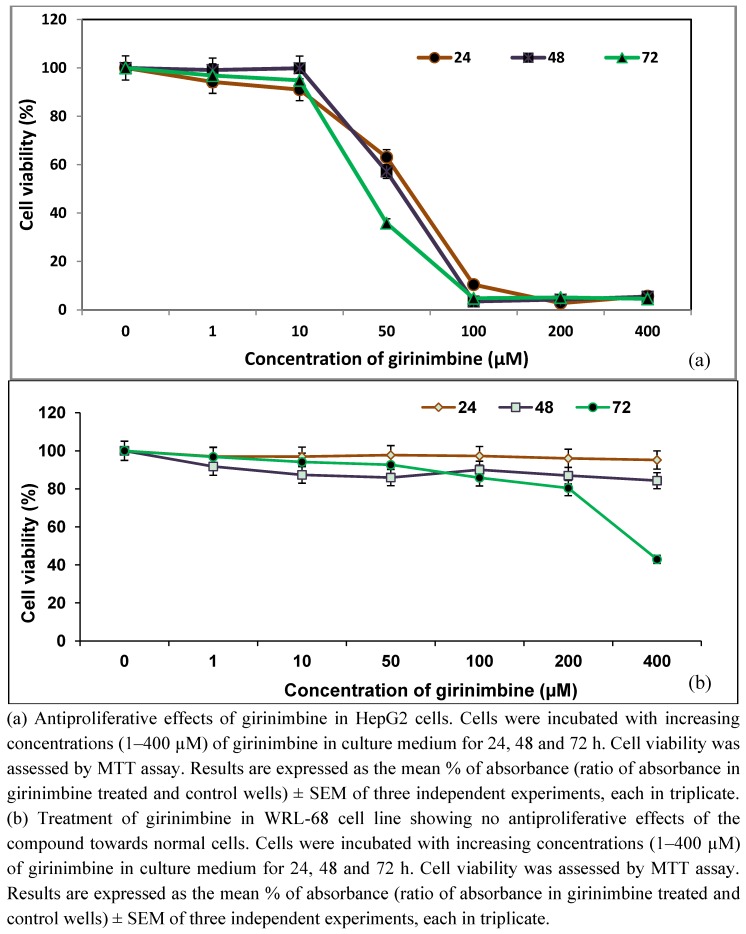
(**a**) MTT Assay in HepG2 cells; (**b**) MTT assay in WRL-68 cells.

As shown in Figure 2a, girinimbine inhibited the proliferation of HepG2, human HCC cells *in vitro* in a dose- and time-dependent manner. Although no apparent effect on cell viability was observed at lower concentrations (1 and 10 µM), at higher concentrations (100, 200, and 400 µM), girinimbine was capable of decreasing the viability of HepG2 cells in 24, 48 and 72 h with IC_50_ of 61 ± 2.3 µM, 56 ± 3.6 µM, and 40 ± 2.7 µM respectively. Paclitaxel (used as positive control), a commercially available anticancer agent against broad range of cancers [20] including HCC, produced an inhibitory effect on HepG2 cells with an IC_50_ value of 0.031 ± 0.0022 µM. In contrast to these results, there was no evidence of significant antiproliferative effect on normal liver cells, WRL-68 even at concentrations of 200 µM (Figure 2b).

### 2.2. Girinimbine Increased LDH Release from HepG2 Cells

**Figure 3:**
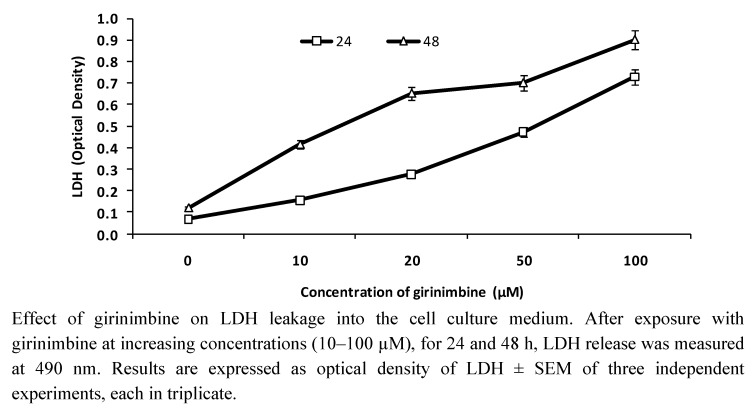
LDH Leakage Assay.

The plasma membrane integrity was evaluated by measuring the release of LDH in the culture medium. After treatment of HepG2 cells with increasing concentrations of girinimbine (10–100 µM) for 24 and 48 h, the results were in agreement with that of the MTT assay. That is, there was significant cytotoxicity observed with the increase of LDH leakage in both concentration- and time-dependent manner (Figure 3).

### 2.3. Girinimbine Induces Morphological Changes in HepG2 Cells Prior to Cell Death

**Figure 4:**
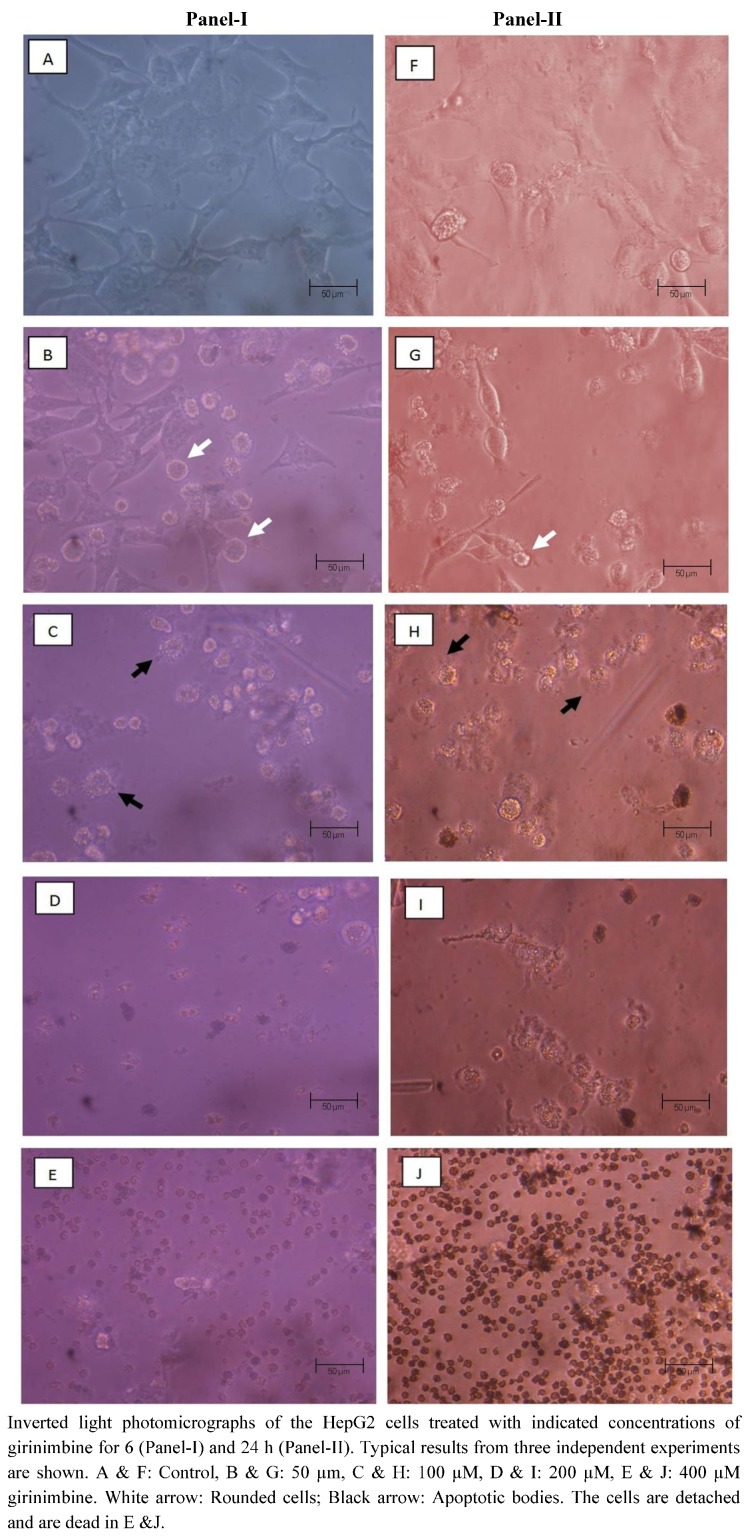
Inverted light photomicrographs of girinimbine treated HepG2 cells.

**Figure 5:**
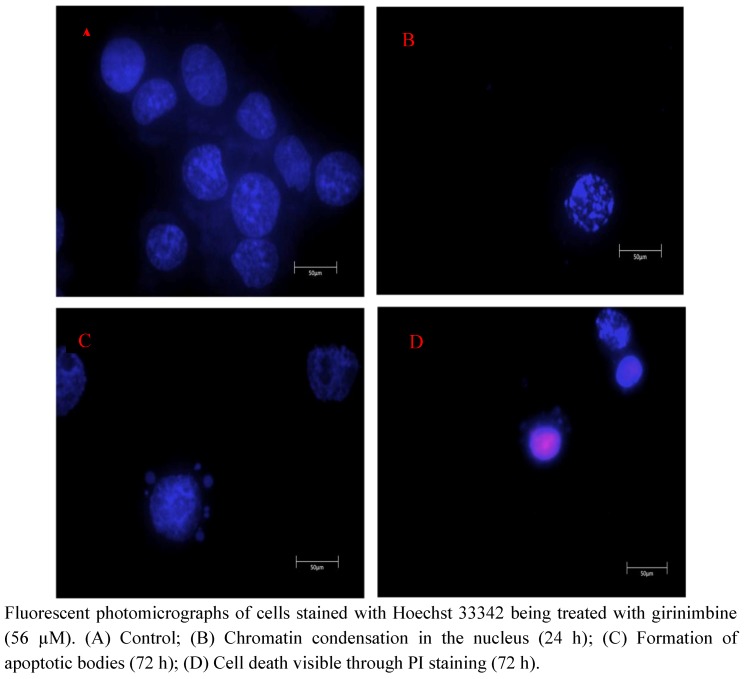
Hoechst 33342 Fluorescent photomicrographs.

Morphological features of girinimbine-induced cell death were studied using an inverted light microscope. Early effects like rounding up of cells and blebbing of the plasma membrane were evident (Figure 4). These were even visible at 6 h of girinimbine treatment. With the high concentration of 400 µM there were almost no live cells at 6 h. Staining the cells with Hoechst 33342 showed the typical features of apoptosis such as chromatin condensation, and formation of apoptotic bodies. Generally, the affected nuclei appeared smaller; some had peripherally condensed or clumped chromatin whereas others had fragmented nuclear chromatin (Figure 5). Blebbing was more prominent at 72 h post-treatment of 56 µM girinimbine. The observations suggest that the progression of cell death is both concentration- and time-dependent.

### 2.4. Girinimbine Showed Biochemical Features of Apoptosis

**Figure 6:**
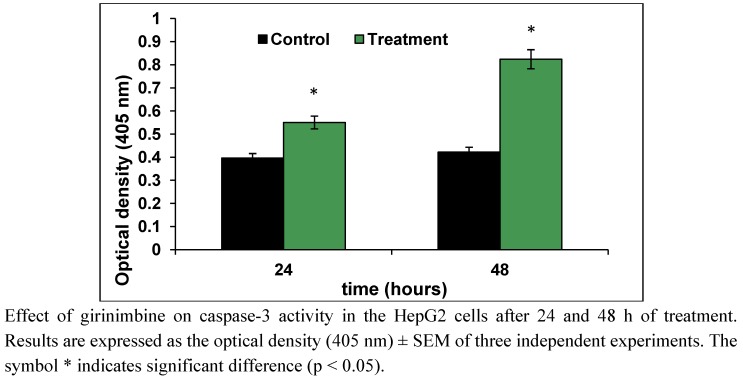
Caspase-3 activity of girinimbine treated HepG2 cells.

**Figure 7:**
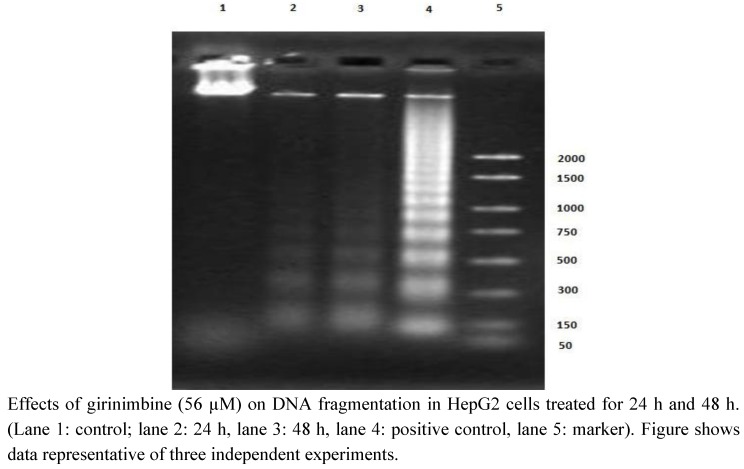
DNA laddering in girinimbine treated HepG2 cells.

Caspase-3 is the major executor caspase at the downstream of the apoptosis cascade, which is activated by other initiators and upstream caspases. There was a two-fold increase in caspase-3 activity in the HepG2 cells at 48 h after girinimbine treatment (Figure 6). The DNA from apoptotic cells on analysis with agarose electrophoresis produced a characteristic DNA ladder which is indicated to be a biochemical hallmark of apoptosis [21]. The results in Figure 7 clearly indicate that girinimbine is capable of inducing time-dependent increase in 180 bp multimetric bands.

### 2.5. Girinimbine Induces G_0_/G_1_-Phase Arrest in HepG2 Cells

**Figure 8:**
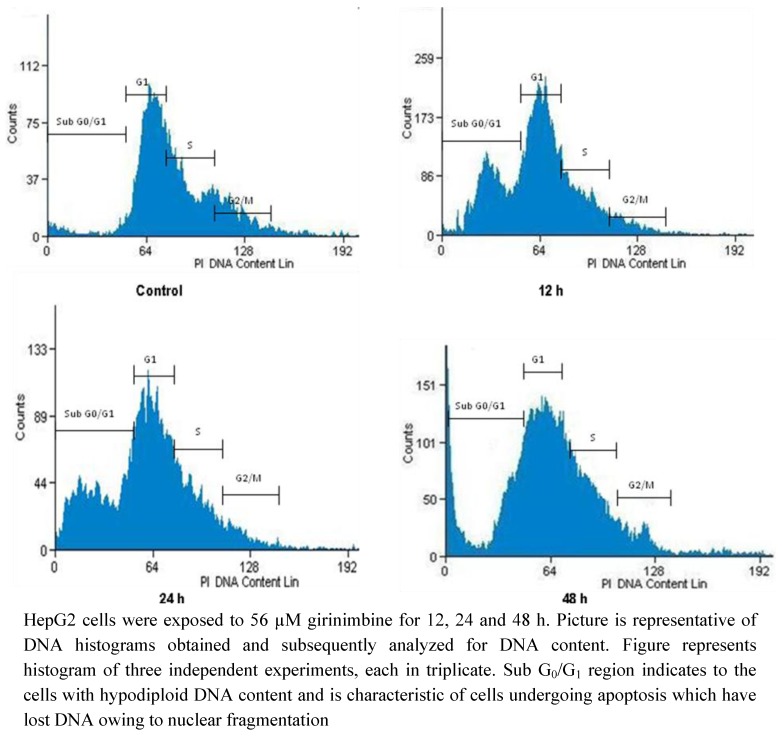
Flow cytometry analysis of girinimbine-treated HepG2 cells.

To look into the mechanisms leading to the loss of cell proliferation by girinimbine, flow cytometric analysis of cell cycle was conducted to know if there is any cell cycle arrest occurring. Cells treated with 56 µM girinimbine showed cell cycle arrest at the G_0_/G_1_-phase. For example, girinimbine treatment for 24 h increased the percentage of cells in the G_0_/G_1_-phase from 67.86% to 72.09%, with a parallel reduction in the percentage of cells in the S- and G_2_/M-phase. The percentage of HepG2 cells in Sub G_0_/G_1_ was also increased (from about 3.81% to 10.34%) during girinimbine treatment. The results (Figure 8 & Table 1) suggested that girinimbine induced apoptosis and inhibited cell proliferation of HepG2 cells via G_0_/G_1_-phase arrest of the cell cycle.

**Table 1:** Distribution of girinimbine treated HepG2 cells in each phase of cell cycle.

	Sub G_0_/G_1_	G_0_/G_1_	S	G_2_/M
**Control**	3.81 ± 0.41	67.86 ± 7.8	8.93 ± 0.1	19.4 ± 1.2
**12 h**	6.56 ± 0.53 *	69.63 ± 6.44 *	6.77 ± 0.21 *	17.04 ± 1.5 *
**24 h**	7.62 ± 0.19 *	72.09 ± 8 .1 *	5.21 ± 0.43 *	15.08 ± 2.1 *
**48 h**	10.34 ± 0.84 *	75.32 ± 7.9 *	5.74 ± 0.4 *	8.6 ± 0.9 *

Induction of G_0_/G_1_ arrest in the cell cycle progression of HepG2 cells by girinimbine. ‘*’ Indicates a significant difference (p < 0.05). Table shows data ± SEM of three independent experiments, each in triplicate.

## 3. Discussion

Even though the plant food and medicinal herb *Murraya koenigii* [22] has been used for centuries as a folk medicine for various cancers, the studies on potent pure compounds derived from it are limited. Previously, girinimbine, one of the carbazole alkaloid from this herbal medicine has been found to inhibit cancer cell proliferation [17,18] and promote apoptosis in human cancer cell lines [11,17,18]. For instance, in a study with mammalian tsFT210 cells girinimbine was shown to induce cell cycle arrest and promote apoptosis [11]. In K562 and HCT-15 cells, girinimbine treatment produced significant DNA ladder bands and typical morphological changes [17,18]. However, there could be more cancer cells that this compound could induce apoptosis. In this study we focused to evaluate the effects of girinimbine on the growth and death of human HCC cell line, HepG2. The results from this study revealed that girinimbine has significant anticancer activity against HCC, HepG2 via induction of cell cycle arrest. The presence of apoptosis in girinimbine treated HepG2 cells was confirmed by chromatin condensation assay, caspase-3 like activity, and DNA fragmentation, whereas the cell cycle evaluation demonstrated G_0_/G_1_ phase arrest. 

The cytotoxic potential of girinimbine is already established in various cancer cell lines including HCT-15, K562, HL-60, HT-29, MCF-7 and HeLa [17,18,23]. Although there was less cell inhibitory activity than the standard drug, paclitaxel (0.031 ± 0.0022 µM), girinimbine showed significant inhibitory activity in HepG2 cells. The proliferation of HepG2 cells were found to be reduced not only dose-dependent but time-dependently too. Such a mode of inhibition was similar to that shown by a number of other natural products. For example, mahanine, a carbazole alkaloid, decreased cell viability of U937 cells both dose and time dependently [24]. From the previous data we believe that the antiproliferative property may be a basic feature of carbazole alkaloids. For instance, the carbazole alkaloids derived from *M. koenigii-* mahanimbine, murrayafoline A and S-benzyldithiocarbazate has exhibited antiproliferative effect on CEMss cells [25]. 

Simultaneously, girinimbine treatment increased the LDH release which is similar to another natural anticancer agent, resveratrol, that demonstrated dose- and time-dependent cytotoxicity in U251 cells [26]. In the current study there was significant cytotoxicity evident from the inverse correlation of decreased cell viability and increased cell membrane damage. The loss of cell viability is thus attributed to the cytotoxic effect of the compound [27]. However, like clausine-B, yet another carbazole alkaloid [28], the inhibitory effect of girinimbine was cancer cell specific with no effect on normal liver cells. Thus the current study identified that girinimbine was capable to elicit cytotoxicity with cancer specificity. 

One of the intriguing findings of the current study is that the cell death induced by girinimbine is through apoptosis, an active programmed cell death that avoids eliciting inflammation [28]. Previously some carbazole alkaloids including girinimbine had shown the potential to induce apoptosis in cancer cells [17,18,30,31]. Strongly supporting our hypothesis, girinimbine treated cells showed typical morphological patterns of apoptosis as described by Kerr, Wyllie and Currie in 1972 [32]. We observed the onset of apoptosis marked by the cell shrinkage followed by pyknosis, which is the most unique feature of apoptosis. Later on, in addition to plasma membrane blebbing, nuclear fragmentation and formation of apoptotic bodies were visible. These findings were further validated by the two important biochemical hallmarks of apoptosis- caspase-3 like activity [33] and the DNA ladder formation [34]. In line with the findings of Ito and fellow researchers [35] where the carbazole alkaloids mahanine, pyrayafoline-D and murrafoline-I showed time dependent increase in caspase-3 activity in HL-60 cells, girinimbine also demonstrated increase in caspase-3 activity in HepG2 cells with time. Being a downstream caspase of both extrinsic and intrinsic pathways of apoptosis [36] and responsible partially or totally for cleavage of many key proteins resulting in DNA-fragmentation and other morphological changes [33], caspase-3 is very crucial for apoptosis induction. Therefore, we next focused on DNA-fragmentation and found that girinimbine treatment led to fragmentation of DNA in HepG2 cells.

In order to further elucidate the mechanism of apoptosis the cell cycle analysis was performed. Previously two carbazole alkaloids mahanimbine and murrayafoline-A have been shown to induce apoptosis by their ability to arrest cells in the G_0_/G_1_ phase of the cell cycle in CEMss [25]. In this respect there is accumulating evidence that carbazole alkaloids can naturally inhibit the cell cycle process [25,31]. Hence, on conducting the flow cytometric evaluation of girinimbine treated HepG2 cells, the hypothesis of apoptosis induction via cell cycle arrest was confirmed. Different from clausine-E, a carbazole alkaloid that arrested different colorectal cancer cell lines at G_2_/M phase [37], girinimbine arrested HepG2 cells at the G_0_/G_1_ phase. 

On the whole, the results of the present study demonstrate the antiproliferative and apoptosis- inducing properties of girinimbine, which are consistent with those of other carbazole alkaloids. The previous data indicates the antiproliferative effects of girinimbine on human colon carcinoma cell line, HCT-15 [17] and human myelogenous leukemia cell line, K562 [18], proving that anticancer activity is an integral part of the basic nature of girinimbine. There is also accumulating evidence for the cell cycle inhibitory effects of carbazole alkaloids from previous literature. For example, ellipticine is known to induce apoptosis via G_2_/M phase cell cycle arrest in human breast cancer cells, MDA-MB-231 and MCF-7 [30,31]. Similarly, as evident from the results, girinimbine ultimately led to apoptosis through G_0_/G_1_ phase arrest.

On the basis of the observations mentioned in this report, we have demonstrated for the first time that girinimbine acts as an anticancer agent by induction of dose and time dependent apoptosis in HepG2 through G_0_/G_1_-phase cell cycle inhibition. Our findings suggest that girinimbine may be a potent molecular target for medical treatment of HCC. The results of this study warrant further in depth *in vitro* and *in vivo* studies. The positive outcomes of such studies could be strong basis for developing girinimbine as a novel chemotherapeutic agent for HCC intervention. 

## 4. Experimental

### 4.1. Drugs and Reagents

Girinimbine used in this investigation was isolated from roots of *Murraya koenigii* by Abubakar and co-workers [19]. Root of *M. koenigii* was collected from Sik, Kedah, Malaysia in 2005. The experimental work on extracts of the plant to afford girinimbine and its spectroscopic data has been reported previously [19]. Girinimbine stock solution was at a concentration of 10 mg/mL in dimethyl sulfoxide (DMSO) and the final concentration of DMSO was 0.1% (v/v). Different concentration of the sample was prepared with serial dilution. DMSO (0.1%) was used as a control. Materials for MTT assay, LDH assay, and Hoechst 33342 assay were purchased from Sigma-Aldrich, Malaysia. Kit for Caspase-3 assay was obtained from BioSource International, Inc., (Camarillo, CA, USA), RNase A and Suicide-Track™ DNA Ladder Isolation kit were obtained from Calbiochem (San Diego, CA, USA). All other reagents used were of analytic grade. 

### 4.2. Cell Culture

HepG2 and WRL-68 cells were obtained from American Type Culture Collection (Rockville, MD, USA). Both the cells were maintained at 37 °C in a humidified incubator containing 5% CO_2_ and grown in RPMI 1640 medium supplemented with 10% fetal bovine serum and 1% pen/strep.

### 4.3. Cell Viability Assay

The percentage of growth inhibition was determined by MTT assay. MTT is a yellow water-soluble dye that is reduced in viable cells to an insoluble purple coloured product, MTT-formazan (3-[4,5-dimethylthiazol-2-yl]-3,5-diphenylformazan) by the mitochondrial enzyme succinate dehydrogenase. In brief, cells were seeded in 96-well microplates at a density of 1 × 10^5^ cells/mL. After 24 h incubation, the cells were treated with various concentrations of girinimbine (1,10,50,100, 200 and 400 µM) for 24, 48 and 72 h. After treatment, 20 μL of MTT solution (5 mg/mL in PBS) was added in each well and incubated for 4 h. Subsequently, the media was removed and the formazan crystals were dissolved with 100 µL DMSO. Finally the absorbance was measured at 550 nm in a microplate reader (TECAN, SunriseTM, Männedorf, Switzerland). Cell viability was measured as the percentage of absorbance compared to control. The 50% inhibitory concentration (IC_50_) value, defined as the amount of girinimbine that inhibits 50% of cell growth, was calculated from concentration–response curves. Three independent experiments performed in triplicate were used for these calculations. 

### 4.4. LDH Release Assay

Plasma membrane integrity was measured by determining the extent of lactate dehydrogenase (LDH) leakage from cells as measured by the LDH Assay Kit (TOX7, Sigma-Aldrich, Malaysia) following the manufacturer's instructions. In brief, cells were seeded in 96-well microplates at a density of 1 × 10^5^ cells/mL. After 24 h incubation, the cells were treated with various concentrations of girinimbine (10, 0, 50 and 100 µM) for 24 and 48 h. After treatment, the cells were centrifuged and the 50 µL of supernatant was collected into another 96-well microplate. To each well 100 µL Lactate Dehydrogenase Assay Mixture was added and incubated in dark for 30 min in room temperature. The reaction was stopped with 1 N HCl and measured the absorbance at a wavelength of 490 nm in a microplate reader (TECAN, SunriseTM, Männedorf, Switzerland). Three independent experiments performed in triplicate were used for these calculations. 

### 4.5. Evaluation of Morphology by Light Microscopy

HepG2 cells with a density 1 × 10^5^ cells/mL were plated in 6-well plates and the next day it was treated with girinimbine at increasing concentrations (50, 100, 200 and 400 µM). The cells were observed for the morphological characteristics under phase contrast inverted microscope. Morphological changes like rounding up of cells, plasma membrane blebbing and cell detachment were observed. 

### 4.6. Chromatin Condensation Assay

For detection of apoptotic cells, apoptotic nuclear morphology was observed by staining with Hoechst 33342. HepG2 cells at a density 1 × 10^5^ cells/mL were seeded on a 6-well culture plate. Cells were treated with 56 µM of girinimbine for 24, 48, and 72 h. After treatment, cells were collected and washed with cold PBS. The cells were later on stained with 10 µL Hoechst 33342 (1 mM) and 5 µL PI (100 µg/mL) and then observed under a fluorescence microscope (Lieca attached with Q-Floro Software).

### 4.7. Colourimetric Assay of Caspase-3

For the confirmation of apoptosis, the colourimetric protease assay of caspase-3 was performed using commercial kit (ApoTarget kit, BioSource International, Inc.). HepG2 cells with density 1 × 10^5^ cells/mL were treated with 56 µM girinimbine for 24 and 48 h. Untreated cells at 48 h was used as control. The cells were then lysed by using 50 µL of chilled cell lysis buffer and incubated on ice for 10 min. The cell lysates were centrifuged at 10,000 × g for 1 min. The supernatant was collected and 50 µL of 2× reaction buffer (containing 10 mM DTT) were added. To this 5 µL of DEVD-pNA (caspase-3-substrate) was added and incubated in dark at 37 °C for 1 h. After the incubation, the samples were read at 405 nm in a microplate reader (TECAN, SunriseTM, Männedorf, Switzerland). Data was presented as optical density (405 nm; mean SD).

### 4.8. DNA Laddering

HepG2 cells were seeded at a density 1 × 10^5^ cells/mL in culture flask and incubated for 24 h. The cells were then treated with girinimbine (56 µM) for 24 and 48 h. Suicide-Track™ DNA Ladder Isolation kit (Calbiochem) was used for performing the DNA laddering assay. The principle involves detecting the cytoplasmic histone-associated DNA fragments (mononucleosome and oligonucleosomes) formed during apoptosis. Briefly, cells were trypsinized and centrifuged at 1,000 rpm for 10 min (both adherent cells and supernatants). The pellet was gently resuspend in 55 μL of solution #1 (kit component). It was then added with 20 μL of solution #2 (kit component) and was incubated for 60 min. Afterwards 25 μL of solution #3 (kit component) was added, gently mixed and incubated at 50 °C for 3 h. The DNA was precipitated with the kit reagents provided and dissolved in 50 μL of resuspension buffer. For detecting the DNA ladder, the extracted DNA samples were run on a 1.5% agarose gel in Tris–acetic acid–EDTA buffer. HL-60 cells induced to undergo apoptosis with Actinomycin D, which was supplied with the kit manufacturer was used as positive control. After electrophoresis, the gel was stained with ethidium bromide (Gibco BRL Co. Ltd., Paisley, Scotland), visualized with a UV light transilluminator, and photographed.

### 4.9. Flow Cytometric Analysis of DNA Cell Cycle

HepG2 cells were seeded at a density 1 × 10^5^ cells/mL in culture flask and incubated for 24 h. The cells were then treated with girinimbine (56 µM) for 24 and 48 h. After incubation, the cells were centrifuged at 1,000 rpm for 10 min. The pellets were washed twice with PBS to remove any remaining media. In order to restore the integrity, cells were fixed and then flow cytometric analysis was performed. Briefly, cell pellets were fixed by mixing 500 µL of 70% cold ethanol and kept at −20 °C overnight. The cells were then centrifuged at 1,000 rpm for 10 min and washed twice with PBS. After the last wash, 20 µL of RNase A (10 µg/mL) and 2 µL of propidium iodide (PI) (2.5 μg/mL) were added to the fixed cells for 30 min at dark on ice. The DNA content of cells was then analyzed with a Dako flow cytometer equipped with an argon laser (Cyan ADP, Dako, Denmark), where the analysis was performed using Summit V4.3 software. The fluorescence intensity of Sub G_0_/G_1_ cell fraction represents apoptotic cell population.

## 5. Conclusions

The anticancer activity of girinimbine, the carbazole alkaloid from *M. Koenigii*, on hepatocellular carcinoma was studied by various methods including cell viability assay, morphological analysis, DNA laddering, flow-cytometric analysis and caspase-3 activity. Results from all the experiments suggested that girinimbine could significantly inhibit proliferation and induce apoptosis in a human HepG2 cells without any inhibitory effect on normal liver cell line.
